# Imaging the Directed Transport of Single Engineered RNA Transcripts in Real-Time Using Ratiometric Bimolecular Beacons

**DOI:** 10.1371/journal.pone.0085813

**Published:** 2014-01-15

**Authors:** Xuemei Zhang, Allison L. Zajac, Lingyan Huang, Mark A. Behlke, Andrew Tsourkas

**Affiliations:** 1 Department of Bioengineering, University of Pennsylvania, Philadelphia, Pennsylvania, United States of America; 2 Cell Biology and Physiology, University of Pennsylvania, Philadelphia, Pennsylvania, United States of America; 3 Integrated DNA Technologies, Inc., Coralville, Iowa, United States of America; Stanford, United States of America

## Abstract

The relationship between RNA expression and cell function can often be difficult to decipher due to the presence of both temporal and sub-cellular processing of RNA. These intricacies of RNA regulation can often be overlooked when only acquiring global measurements of RNA expression. This has led to development of several tools that allow for the real-time imaging of individual engineered RNA transcripts in living cells. Here, we describe a new technique that utilizes an oligonucleotide-based probe, ratiometric bimolecular beacon (RBMB), to image RNA transcripts that were engineered to contain 96-tandem repeats of the RBMB target sequence in the 3′-untranslated region. Binding of RBMBs to the target RNA resulted in discrete bright fluorescent spots, representing individual transcripts, that could be imaged in real-time. Since RBMBs are a synthetic probe, the use of photostable, bright, and red-shifted fluorophores led to a high signal-to-background. RNA motion was readily characterized by both mean squared displacement and moment scaling spectrum analyses. These analyses revealed clear examples of directed, Brownian, and subdiffusive movements.

## Introduction

RNA expression is a dynamic process that is regulated by a wide variety of intracellular factors including proteins, other RNA species, and small molecules. In addition to having direct effects on global RNA levels, these factors can also influence RNA trafficking and distribution, allowing for subcellular regulation. Due to the complex and diverse nature of RNA regulation, it is apparent that there is a need to acquire both a spatial and temporal profile of RNA expression at the single cell level to unravel the relationship between RNA processing and cell function. This has led to the development of several approaches that are capable of imaging individual RNA transcripts in single living cells, in real-time [1]. The earliest single-molecule RNA imaging studies involved microinjecting cells with RNA that was transcribed and fluorescently labeled *in vitro* [2]. This approach exhibits sufficient signal-to-background for single molecule imaging, due to the presence of multiple fluorophores per RNA transcript and the ability to remove any fluorescent labels not associated with the RNA. Microinjected RNA transcripts have been successfully imaged in live cells from various species, including *Drosophila* [3], mice [2], rat [4], monkey [5] and *Xenopus* [6]. However, a major concern associated with this technique is that since the RNA is not transcribed and processed intracellularly, any mechanisms observed may not reflect true RNA behavior.

Perhaps, the most widely adopted approach to detect individual RNA transcripts in living cells utilizes a green fluorescent protein (GFP) fused to the coat protein of bacterial phage MS2 (GFP-MS2) [7], [8]. This fusion complex is stably introduced into cells that co-express an engineered RNA construct with tandem repeats of the MS2 binding site in the 3′-UTR. The high local concentration of GFP that results from binding to multiple MS2 sites on each RNA transcript results in a discrete fluorescent spot that represents a single RNA transcript. The GFP-MS2 system was first used to monitor *ASH1* mRNA particles in live yeast [7]. However, since this groundbreaking study, the GFP-MS2 system has facilitated the analysis of mRNA localization and trafficking in a variety of organisms, including yeast [7], *Drosophila* [9]–[11], and mammalian cells [12]–[16]. Using the GFP-MS2 approach, it was demonstrated that RNA molecules in the cytoplasm of living cells can be actively transported along cytoskeletal cables, they can be static and anchored on either microtubules or microfilaments, or they can undergo random or corralled diffusion [14]. Evidence of local regulation has also been revealed. Specifically, when β-actin mRNA was labeled with MS2-GFP it exhibited unrestricted Brownian motion at the cell's leading edge but restricted Brownian motion in the perinuclear region [17]. In contrast, RNA molecules in the nucleus exhibited simple diffusion [18].

A significant limitation of the GFP-MS2 system is the high background signal that stems from the GFP-MS2 fusion proteins that are not bound to target RNA. This makes this system sensitive to the relative and total level of target RNA expression and GFP-MS2 expression. One approach that has been taken to minimize this background signal involves redirecting the unbound GFP-MS2 to a subcellular compartment that is removed from the site of interest [14]. Specifically, cytoplasmic RNA can be more readily visualized if the GFP-MS2 fusion protein also contains a nuclear localization sequence that drives unbound GFP into the nucleus. Thus, the GFP-MS2 is only in the cytoplasm when bound to the target RNA. Conversely, nuclear RNA is more clearly visualized if the unbound GFP-MS2 fusion proteins are trafficked to the cytoplasm via a nuclear export sequence.

Recently, several groups have also sought to improve upon the GFP-MS2 system by adopting alternative combinations of RNA binding proteins and their respective RNA targets (e.g. PUMILIO homology domain/nanos response element, bacteriophage lambda-N peptide/boxB, etc.) [19]–[21] and by using protein fragment complementation (PFC) to restrict fluorescence to sites of RNA binding [21]. With PFC, proper folding of the GFP is not achieved until the two GFP fragments are held in close proximity by binding adjacent sites on target RNA. It has also been suggested that fluorescence resonance energy transfer (FRET) may be used to improve signal-to-background, whereby a FRET signal is only generated upon the binding of fluorescent protein-FRET pairs to adjacent sequences on the target RNA [8]. While these techniques may improve the signal-to-background owing to a much lower background signal, they do suffer from a significant reduction in brightness. This could explain why PFC and FRET-based techniques have yet to be widely used for the imaging of single RNA transcripts.

Another fluorescent probe that has been widely adopted for the imaging of RNA in living cells is the molecular beacon (MB) [22]. MBs are oligonucleotide-based probes that are labeled at one end with a fluorescent reporter and at the other end with a quencher. In the absence of complementary nucleic acid targets, the MB forms a hairpin structure, which serves to hold the fluorescent reporter and quencher in close proximity. In this configuration, the fluorescence is quenched. In the presence of complementary nucleic acids, hybridization between the central loop of the MB and the target leads to unfolding of the stem and separation of the fluorescent reporter and quencher. In this configuration, fluorescence is restored. Although a number of studies have shown that MBs can be used to detect RNA in single living cells [23]–[27], there is growing evidence that the sensitivity of RNA detection is significantly hampered by the sequestration of MBs into the nucleus, where they emit false-positive signals [28]–[32], and by large variations in cellular fluorescence that result from heterogeneous intracellular delivery [33]. Nonetheless, imaging of single transcripts has still been achieved by binding up to 96 molecular beacons to engineered RNA constructs with complementary tandem repeats in the 3′-UTR [34], [35].

**Figure 1:**
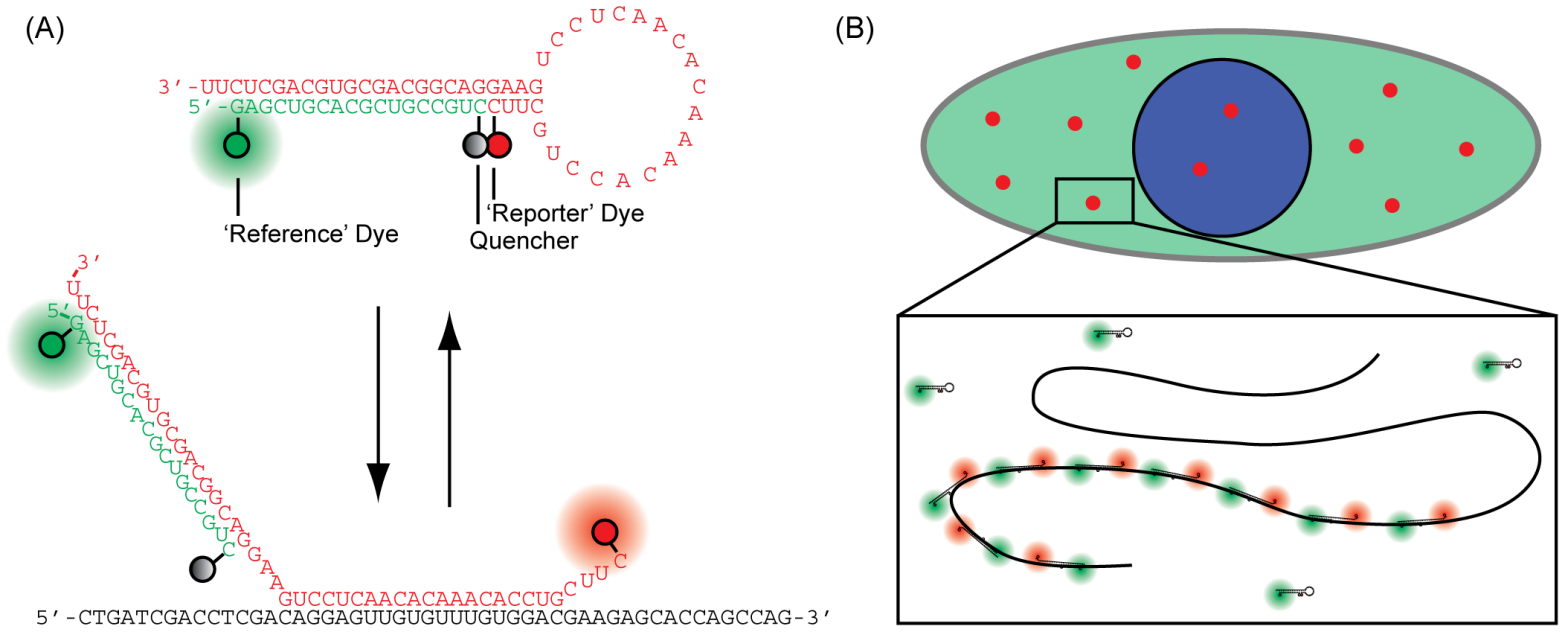
Schematic of RBMBs and the methodology used to detect individual RNA transcripts in living cells. (A) RBMBs are hairpin-forming oligonucleotide probes that are labeled with a reporter dye, quencher, and reference dye. The close proximity of the reporter dye and quencher in the absence of target RNA results in a low fluorescent state. Upon hybridization to complementary RNA, the fluorescent dye and quencher are forced apart, resulting in the restoration of fluorescence. The reference dye remains unquenched regardless of the conformation of the RBMB. The double-stranded domain with a 3′-UU overhang drives nuclear export. (B) To detect individual RNA transcripts, cells were engineered to stably express RNA with 96-tandem repeats of the RBMB target site in the 3′-untranslated region. Binding of up to 96 RBMBs to each RNA transcript results in discrete bright fluorescent spots that can be readily visualized and tracked in real-time by wide-field fluorescence microscopy.

Recently, we developed a new probe for imaging RNA in living cells, ratiometric bimolecular beacons (RBMBs; Figure 1A), to overcome the limitations of conventional MBs and improve the signal-to-background [36]. Similar to MBs, RBMBs form a hairpin structure and elicit an increase in reporter fluorescence upon hybridization to complementary RNA. However, RBMBs are also designed to possess an 18-base pair double-stranded domain with a 3′-UU overhang and an unquenched reference dye. The unique structure of the RBMB facilitates nuclear export, which dramatically reduces the level of false-positive signals that are detected for at least 24 hours, compared with conventional MBs. The reference dye allows for measurements of reporter fluorescence to be adjusted for cell-to-cell variability in RBMB delivery, which allows for more precise measurements of RNA hybridization. We have demonstrated that RBMBs can be used to image individual RNA transcripts in single living cells, when the target RNA is engineered to contain as few as 4-RBMB binding sites [37]. Moreover, RNA expression can be accurately quantified at both the single molecule level and at the single cell level. Measurements of gene expression can be acquired within 30 minutes following the delivery of RBMBs and the methodology is fairly insensitive to RBMB concentration and target RNA levels, since fluorescence from unbound RBMBs is efficiently quenched.

In this study, we show for the first time that RBMBs can be used as a new tool to study the directed transport of single RNA molecule in living cells. RBMBs were introduced into adherent cells stably expressing *GFP* RNA with 96-tandem repeats of the RBMB target sequence within the 3′-untranslated region. Upon hybridization, the RNA transcripts were visualized as discrete, bright fluorescent spots by wide-field fluorescence microscopy with high signal-to-background (Figure 1B). The transcripts were observed within the nucleus and cytoplasm and their movements were readily analyzed by mean squared displacement analysis and moment scaling spectrum analysis. Our findings suggest that RBMBs represent a new, robust, and powerful tool for the analysis of single engineered RNA transcripts in living cells.

## Materials and Methods

### Synthesis and design of RBMBs

RBMBs are composed of two 2′-O-methyl RNA oligonucleotides that are hybridized together. One of the oligonucleotides is labeled with a CF640R (Biotium, Hayward, CA) reporter dye at the 5′-end and has the sequence: 5′-CF640R-mCmUmUmC mGmUmC mCmAmC mAmAmA mCmAmC mAmAmC mUmCmC mU mGmAmAmG mGmAmC mGmGmC mAmGmC mGmUmG mCmAmG mCmUmC mUmU-3′. Self-complementary domains, which drive the formation of the hairpin structure, are underlined. The second oligonucleotide is labeled at the 5′-end with an Alexa Fluor 750® reference dye (AF750) and at the 3′-end with an Iowa Black RQ-Sp quencher (IBRQ, Integrated DNA Technologies, Coralville, IA). The sequence of the second oligonucleotide is: 5′-AF750-mGmAmG mCmUmG mCmAmC mGmCmU mGmCmC mGmUmC-IBRQ-3′. To form RBMBs, the two oligonucleotides are hybridized at a molar ratio of 1∶1.5 RBMB1∶RBMB2 in phosphate buffer (48 mM K_2_HPO_4_, 4.5 mM KH_2_PO_4_, 14 mM NaH_2_PO_4_, pH 7.2) at room temperature overnight. A slightly higher concentration of RBMB2 is utilized to ensure complete hybridization of RBMB1. The presence of unbound RBMB1 in cells can create an unwanted background signal. Excess RBMB2 is also easier to distinguish from hybridized RBMBs via size exclusion chromatography, than RBMB1. The hybridized RBMB is purified using a Superdex 75 prep grade column (GE healthcare, Piscataway, NJ) and concentrated using a Microcon YM-10 centrifugal device (10, 000 MW cutoff, Millipore, Billerica, MA). The final concentration of the RBMBs was determined using a Cary 100 spectrophotometer (Varian, Palo Alto, CA).

### Cell culture and cell line construction

Human fibrosarcoma cells, HT1080, were purchased from ATCC (ATCC, Manassas, VA), and were cultured in MEM media supplemented with 1% Pen/Strep and 10% fetal bovine serum (FBS). The cells were incubated at 37°C with 5% CO_2_. HT1080-GFP-96mer cells, which stably express *GFP* RNA with 96-tandem repeats of the RBMB target site with the 3′-UTR were generated by infecting HT1080 cells with pLenti-dsGFP-96mer, as previously described [37]. Infected cells were selected for stable genomic integration using 15 mg/mL blasticidin (Invitrogen, Grand Island, NY). The blasticidin-selected cells were further sorted by flow cytometry in the Flow Cytometry and Cell Sorting facility, University of Pennsylvania.

### Cellular delivery of RBMBs

The introduction of RBMBs into cells was achieved by microporation with a Neon transfection system (Invitrogen, Grand Island, NY) as per manufacturer's protocol. Briefly, a cell pellet containing 300,000 cells was resuspended in 11 µL Resuspension Buffer, supplied with the kit, and mixed with 1 µL of 9.6 µM RBMBs (34). The final concentration of RBMBs was 0.8 µM. After microporation, cells were washed 3 times in culture medium (without FBS and Pen/Strep) and seeded on poly-d-lysine (Sigma, St. Louis, MO) coated 8-well chambered coverglass system (Thermo Scientific, Waltham, MA) for ∼1 hr. For studies utilizing the spinning disk confocal system for fluorescence imaging (described below), Hoechst 33342 (Invitrogen) was added at a final concentration of 0.01 µg/µL.

### Image Acquisition

Wide-field fluorescence microscopy was performed using an Olympus IX81 motorized inverted fluorescence microscope equipped with a back-illuminated EMCCD camera (Andor, UK), a SOLA Light Engine (Lumencor, Beaverton, OR) and a CoolLED pE-100 (740 nm, CoolLED, UK). A plan Apo 100× (NA 1.45) oil objective was used. The Stream Acquisition function in Metamorph software (Molecular Devices, Sunnyvale, CA) was used to acquire 150 images in the CF640R channel. Images were acquired using the filter set ET620/60×, ET700/75 m, T660lpxr for CF640R. On occasion, the filter set HQ710/75, HQ810/90, Q750LP was also used to image Alexa 750, to confirm RBMB delivery. Before imaging, cells were equilibrated to 37°C on the microscope stage using a live-cell stage top incubation system (Pathology Devices, Westminster, MD). Imaging was typically performed 1 to 2 hrs after cells were seeded in the wells of the chambered coverglass system.

### Data Analysis

#### Particle Tracking

Local background subtraction was performed on the images using the Rolling Ball Background Subtraction plugin in ImageJ [38]. An in-house tracking algorithm based in ImageJ was used to automatically track RNA punctae. 10 particles exhibiting motion that appeared directed, diffusive, or confined were tracked from 11 movies.

#### Motion Analysis

Trajectories were analyzed using custom MATLAB (Mathworks) code. Mean squared displacement (MSD) analysis of trajectories was performed (Eq. 1) using all pairs, where *r^2^* is mean squared displacement, *n* is time lag, *N* is length of trajectory, and *p* is position.
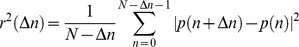
The resulting MSD vs time lag relationship was fit to (Eq2), where *D* is the diffusion coefficient, *t* is the time lag, and *α* is the scaling exponent. The scaling exponent α can be used to define the motion: 0 = static, 1 = diffusive, 2 = directed.
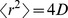
Motion scaling spectrum (MSS) analysis was also performed. MSS analysis is associated with less error than MSD analysis [39] and has previously been applied to classify the motion of CD36 receptors [40] and viruses [41] in live cells. MSD and MSS measurements are expected to be correlated if they are accurately reflecting the motion of the particle. In MSS analysis (Eq 3), the relationship between the displacement over many different moments (*v*) relative to different time lags is analyzed (MSD analysis is the second moment, moments 1–6 were analyzed).
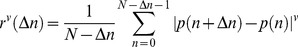
The resulting plot of scaling exponents vs moments has a linear relationship whose slope (S_MSS_) can be used to define motion as confined, diffusive, or directed (0-static, 0.5 = diffusive, 1-directed).

First individual trajectories were analyzed using MSD analysis and separated into three groups (confined, diffusive, and directed) based on their individual α values. MSD analysis was then performed by averaging across all tracks within each group to increase the number of observations at longer time lags and the reliability of the measurement. MSS analysis was performed in the same way on these three groups of tracks.

To measure the speed and displacement of the directed set of trajectories, the position was smoothed over 5 frames (or 1.4 s). The frame-to-frame instantaneous speed was measured for the entire trajectory and the maximum speed per trajectory measured. In addition the net displacement from the start to the end of the track was measured.

## Results and Discussion

In order to image single RNA transcripts in living cells, RBMBs were microporated into HT1080-cells that were engineered to stably express a *GFP* RNA construct with 96-tandem repeats of the RBMB binding site in the 3′-UTR. Cells were imaged approximately 1 to 2 hrs following microporation, to ensure that the cells had time to adhere and spread onto poly-d-lysine coated coverglass. All imaging studies were performed using a live-cell stage-top incubation system operating at 37°C and 5% CO_2_ to ensure physiologic conditions. Individual RNA transcripts within single cells appeared as discrete, bright fluorescent spots via wide-field fluorescence microscopy and RNA movements were readily captured by performing streaming acquisition (Movies S1, S2, S3). Previously we validated that the observed bright fluorescent spots do indeed correspond to individual RNA transcripts and that the observed RNA is completely intact, consisting of both the coding sequence and the tandem repeats in the 3′-UTR [37]. No spots are observed in cells that express GFP RNA that lack the tandem repeats. Here, full-frame images were acquired at a rate of approximately 3.6 frames per second. Approximately 42 seconds of continuous illumination (150 frames) was readily achievable before the fluorescent signals associated with RNA hybridization were significantly reduced due to photobleaching. This timeframe could easily be extended by lowering the intensity of the excitation light at the cost of signal-to-background; however, the shorter time frames were generally adequate, since most RNA transcripts would travel out of the focal plane within tens of seconds.

**Figure 2:**
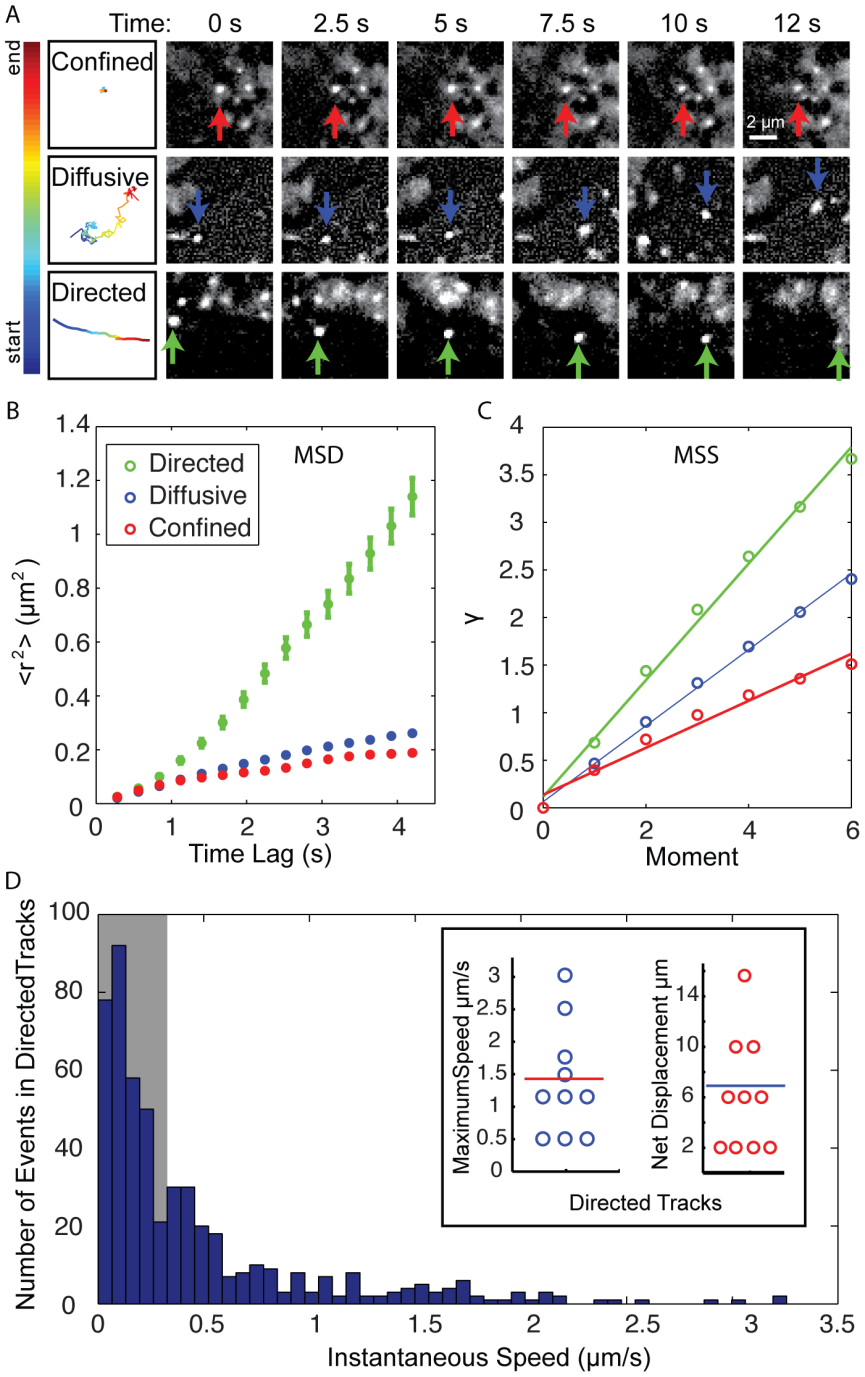
Analysis of RNA motion in living cells. (A) Montage of RNA transcripts classified as confined, diffusive or directed based on mean squared displacement analysis (MSD) analysis. The trajectories (far left) for the transcripts that are indicated by the arrows are color-coded for time/frames. Scale bar: 2 µm. The motion of RNA transcripts was assessed using (B) MSD analysis and (C) motion scaling spectrum (MSS) analysis. The MSD and MSS plots represent 10 tracks per category (mean ± SEM). (D) Analysis of the speed and distance covered by directed particles. After smoothing the position over 5 frames, the instantaneous frame-to-frame velocity was calculated for all directed tracks (N = 10). A histogram of the speed distribution for all tracks is shown with a gray bar indicating speeds below our resolution limit. The inset shows the maximum speed achieved in each directed track (mean = red line, 1.4 µm/s), and the net displacement per track (mean = blue line, 7 µm).

Streaming images revealed that individual RNA transcripts exhibited various types of motion. Some RNA transcripts were confined/subdiffusive, some traveled by random diffusion, and others traveled rapidly along paths by what appeared to be directed transport (Figure 2A and Movies S4). The various types of motions were characterized by mean squared displacement (MSD) and moment scaling spectrum (MSS). According to the MSD analysis, the anomalous diffusion exponent α was determined to be ∼1.4 for transcripts that appeared to be undergoing directed transport, ∼0.9 for transcripts undergoing Brownian motion and ∼0.7 for transcripts with confined motion (Figure 2B). Although the distinction between diffusive and directed motion using measurements of α is subjective, when considered in combination with the rapid speeds that were observed – often >0.5 µm/s – and that these speeds were maintained for several seconds, there is a strong argument for directed transport. This is further supported by the MSS analysis where the slope S_MSS_ was calculated to be 0.6 for transcripts undergoing directed transport, 0.4 for transcripts undergoing Brownian motion, and 0.2 for transcripts that were confined (Figure 2C). Observations of directed transport are consistent with results previously obtained using the GFP-MS2 system, which revealed that even RNAs that lack zip codes can exhibit rapid and directional movements along microtubules and microfilaments [14].

Interestingly, several RNA transcripts were observed to undergo sharp changes in direction (Movie S1), follow tightly curved paths (Movie S5), or switch from directed transport to random diffusion or vice versa (Movies S6). Characterization of RNA transcripts that were categorized as directed exhibited an average RNA velocity of 1.5 µm/sec (Figure 2D). The maximum velocity observed was 3.2 µm/sec (5 frame smoothing). The average distance traveled for individual RNA transcripts was approximately 7 µm with the longest path measured being 16 µm. However, it is likely that these measurements are underestimates since many RNA transcripts traveled through the imaging plane or were subject to photobleaching. Consequently, in many cases only a portion of the path was imaged.

While many of the observations reported here have previously been observed using the GFP-MS2 system [14], RBMBs do offer several notable benefits. In particular, RBMBs exhibit an improved signal-to-background and longer time window for imaging. This stems from the ability to use organic fluorophores. Compared to fluorescent proteins, organic fluorophores are smaller, exhibit better photostability, much higher emissivity, and a wider available spectral range [42]. RBMBs also exhibit a much lower background than the GFP-MS2 system owing to the efficient quenching of fluorescence emanating from unbound RBMBs. The primary disadvantage of using RBMBs is the need for intracellular delivery of the oligonucleotide probe. However, this has been largely circumvented by the introduction of microporation, which has a delivery efficiency close to 100% with little to no effect on cell viability [29]. Miniaturized microporation devices now also offer an opportunity to avoid trypsinizing/dissociating adherent cells for probe delivery [43]. The intracellular lifetime/long-term stability of RBMBs in living cells may also be viewed as a potential limitation, but it has been shown that RBMBs are functionally active for at least 24 hours [36]. Overall, it is believed that RBMBs represent a new, robust tool for imaging single engineered RNA transcripts in living cells that should enable even novice fluorescence microscopy users to study the transport of individual RNA transcripts. Potential applications of this technique include measuring the response of RNA trafficking to various external stimuli, observing unique subcellular localization patterns of target RNA, studying the fate and lifetime of RNA. Moreover, it may also be possible to monitor changes in RNA expression as a function of time and in response to environmental, biological, or chemical perturbations. Therefore, it is envisioned that this technique could provide unique insight into RNA biology.

## Supporting Information

Movie S1 **Representative movie of RNA transcripts moving in living cells.** Individual RNA transcripts appear as discrete fluorescence spots. A transcript undergoing directed transport is indicated by a white arrow. Scale bar: 10 µm.
(MOV)

Movie S2 **Representative movie of RNA transcripts moving in living cells.** Individual RNA transcripts appear as discrete fluorescence spots. A transcript undergoing directed transport is indicated by a white arrow. Scale bar: 10 µm.
(MOV)

Movie S3 **Representative movie of RNA transcripts moving in living cells.** Individual RNA transcripts appear as discrete fluorescence spots. A transcript undergoing directed transport is indicated by a white arrow. Scale bar: 10 µm.
(MOV)

Movie S4 **Montage of RNA motions that have been classified as confined, diffusive or directed based on mean squared displacement analysis (MSD) analysis.** Scale bar: 5 µm.
(MOV)

Movie S5 **Movie of an RNA transcript following a highly curved path.** The RNA track is overlaid over the cell image. Scale bar: 10 µm.
(MOV)

Movie S6 **Movie of an RNA transcript going from directed to diffusion motion.** The RNA track is overlaid over the cell image. Scale bar: 10 µm.
(MOV)
